# Role of SEC14-like phosphatidylinositol transfer proteins in membrane identity and dynamics

**DOI:** 10.3389/fpls.2023.1181031

**Published:** 2023-05-15

**Authors:** Karolin Montag, Rumen Ivanov, Petra Bauer

**Affiliations:** ^1^ Institute of Botany, Heinrich Heine University, Düsseldorf, Germany; ^2^ Center of Excellence on Plant Sciences (CEPLAS), Germany

**Keywords:** lipid binding site, lipid transfer, SEC14, PATELLIN, membrane, phosphatidylinositol, multi-domain, tocopherol

## Abstract

Membrane identity and dynamic processes, that act at membrane sites, provide important cues for regulating transport, signal transduction and communication across membranes. There are still numerous open questions as to how membrane identity changes and the dynamic processes acting at the surface of membranes are regulated in diverse eukaryotes in particular plants and which roles are being played by protein interaction complexes composed of peripheral and integral membrane proteins. One class of peripheral membrane proteins conserved across eukaryotes comprises the SEC14-like phosphatidylinositol transfer proteins (SEC14L-PITPs). These proteins share a SEC14 domain that contributes to membrane identity and fulfills regulatory functions in membrane trafficking by its ability to sense, bind, transport and exchange lipophilic substances between membranes, such as phosphoinositides and diverse other lipophilic substances. SEC14L-PITPs can occur as single-domain SEC14-only proteins in all investigated organisms or with a modular domain structure as multi-domain proteins in animals and streptophytes (comprising charales and land plants). Here, we present an overview on the functional roles of SEC14L-PITPs, with a special focus on the multi-domain SEC14L-PITPs of the SEC14-nodulin and SEC14-GOLD group (PATELLINs, PATLs in plants). This indicates that SEC14L-PITPs play diverse roles from membrane trafficking to organism fitness in plants. We concentrate on the structure of SEC14L-PITPs, their ability to not only bind phospholipids but also other lipophilic ligands, and their ability to regulate complex cellular responses through interacting with proteins at membrane sites.

## Highlights

• SEC14-like phosphatidylinositol transfer proteins (SEC14L-PITPs) can bind phospholipids and other lipophilic ligands.

• The occurrence of multi-domain SEC14L-PITPs in higher eukaryotes including land plants underlines their functional roles.

• Plant SEC14 proteins function as cellular regulators via protein-protein and/or protein-lipid interaction and lipid transfer to achieve chloroplast functioning, cell polarity and development, and to control the response to environmental stimuli and iron nutrition.

## 1 Introduction

Cells are surrounded by membranes, which function as hydrophobic permeable barriers regulating the exchange of molecules and the flow of information. Within the cell, membranes have different compositions resulting in their specific identity and allowing them to fulfill specific tasks (Watson, 2015; Heilmann, 2016; Mamode Cassim et al., 2019). The plasma membrane, for example, is involved in uptake of molecules from the environment, cell-to-cell communication or cell shape changes (Cooper, 2000; Luschnig and Vert, 2014). The thylakoid membrane, on the other hand, is essential for photosynthesis and uses an electron gradient to generate ATP (O'Connor and Adams, 2010). In addition to building the basic membrane backbone, lipids may have regulatory roles (Stevenson et al., 2000). Minor changes in lipid composition and structure can result in major modifications to essential cellular processes (Harayama and Riezman, 2018). Especially the phospholipid composition of a membrane has significant effects on the regulation of cellular and tissue functions (Heilmann, 2016). A crucial group of regulatory phospholipids are the phosphorylated derivatives of phosphatidylinositol (PI), which are the phosphoinositides (PIPs) PI(3)P, PI(4)P, PI(5)P, PI(3,4)P_2_, PI(3,5)P_2_, PI(4,5)P_2_ and PI(3,4,5)P_3_ (Irvine, 2016). PI and PIPs provide cues to membrane identity, although they make up less than 1% of membrane lipids (Simon et al., 2014; Simon et al., 2016; Gerth et al., 2017; Noack and Jaillais, 2020). They are key players controlling growth, development and polarization as well as influencing multiple processes through binding to a great number of interaction partners in adaptation to, e.g., environmental changes (Heilmann, 2016; Roman-Fernandez et al., 2018). It is among the open compelling questions in plant cell biology how membranes are remodeled and controlled and how identity and dynamics of membrane systems are determined, maintained or changed (Roeder et al., 2022). Here, we review one class of peripheral membrane proteins, namely SEC14 domain-containing lipid transfer proteins, that are promising candidates to alter the phospholipid identity and membrane dynamics in their functions as lipid transfer, lipid-binding and membrane-associated proteins with protein-protein interaction capabilities.

## 2 Overview of phosphatidylinositol transfer proteins

Lipids can be distributed in the cell through vesicle-independent trafficking, which includes spontaneous lipid transfer, flip-flop exchange within bilayers, lateral diffusion or single-lipid transfer by lipid-transfer proteins, that act, for example at membrane contact sites (Lev, 2010; Peretti et al., 2019). On the other hand, trafficking of proteins, lipids and other metabolites between cell compartments is achieved by the highly regulated and coordinated vesicular trafficking mechanism, in which macromolecules are transported within membrane vesicles (Tokarev et al., 2013; Goring and Di Sansebastiano, 2017). Generally, single-lipid transfer and membrane vesicular trafficking are controlled by regulatory proteins in response to developmental cues and external stimuli. It is not yet well investigated how this is controlled in plant cells. One group of proteins able to link lipid recognition, metabolism and signaling are phosphatidylinositol transfer proteins (PITPs). PITPs can be clustered in two independent protein families with distinctly separated biological functions (Wirtz, 1991).

The first group is simply named the phosphatidylinositol transfer protein (PITP)-superfamily, defined through its e.g. phosphatidylinositol transfer protein and Lipin/Ned1/Smp2 (PITP/LNS2) domain (InterPro accession number (IPR): IPR031315). Such a domain is thought to promote the exchange of phospholipids at the membrane contact sites of the endoplasmic reticulum (ER) and the plasma membrane by non-vesicular lipid transport (Cockcroft and Raghu, 2018). Proteins containing a PITP/LNS2 domain can be found in mammals, invertebrates and plants (Hsuan and Cockcroft, 2001; Cockcroft, 2012). Defects in PITP proteins can lead to for example neurodegenerative diseases (Hsuan and Cockcroft, 2001; Cockcroft, 2012). In *Arabidopsis thaliana* (Arabidopsis) the PITP/LNS2 domain can for example be found in two phosphatidate phosphohydrolase proteins involved in galactolipid synthesis and necessary to maintain membrane structure by lipid remodeling due to phosphate starvation (Nakamura et al., 2009; Yoshitake et al., 2017). These examples show that PITPs have profound functions in cellular and physiological integrity of higher eukaryotes.

**Figure 1:**
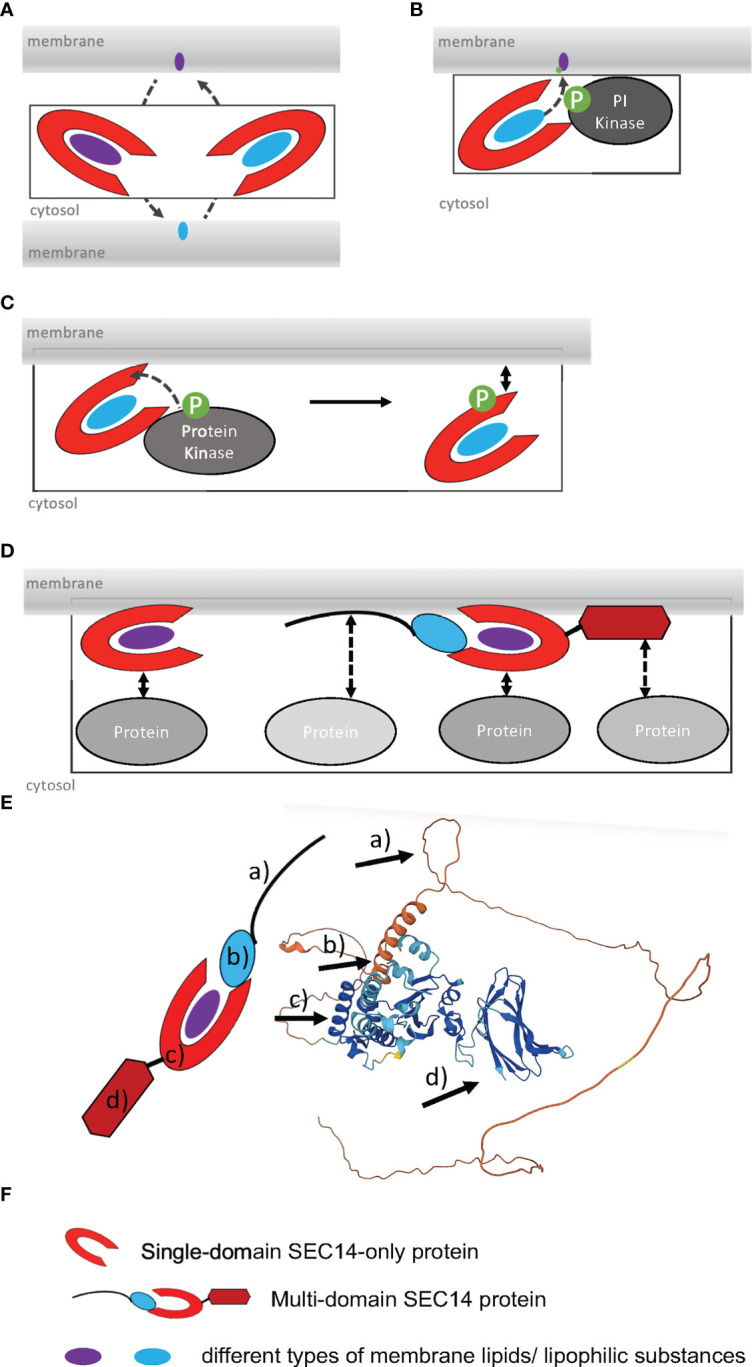
Functions and regulation modes of SEC14L-PITPs. **(A)**, SEC14 protein-mediated lipid transfer and heterotypic exchange of lipids between two membranes. **(B)**, Lipid presentation model, interaction of SEC14 protein with PI kinase and phosphorylation of lipid during transfer (de Campos and Schaaf, 2017). **(C)**, Regulation of membrane binding by phosphorylation of SEC14 domain by a protein kinase. **(D)**, Increase in the number of potential protein-protein interactions of multi-domain versus single-domain SEC14L-PITPs. **(E)**, Alphafold model of the multi-domain SEC14 protein PATL2 (At1g22530). The arrows point to a) intrinsically disordered N-terminal region; b) CTN domain; c) SEC14 domain with lipid-binding site, gate and anchor helices; d) GOLD domain. Alphafold was used, as described (Jumper et al. 2021; Varadi et al. 2021). **(F)**, Symbols used in **(A–D)**.

The second PITP-superfamily and subject of this review is defined through its SEC14 domain (IPR001251), named the SEC14-like phosphatidylinositol transfer protein (SEC14L-PITP)-superfamily. SEC14L-PITPs are able to recognize, bind, exchange and transfer small lipophilic molecules between membranes by non-vesicular transport (**Figures 1A–F**) (Bankaitis et al., 1990; Cleves et al., 1991). Additionally, they are involved in regulation of membrane trafficking within a cell (Bankaitis et al., 2010). SEC14 proteins are found in yeast, plants, invertebrates, and mammals, suggesting a conserved and essential role (Ren et al., 2011; Aravind and Iyer, 2012). In the following sections, we will review their general characteristics, distinguish functions of single- and multi-domain SEC14-PITPs and highlight recent work describing their roles in plants, focusing on *Arabidopsis thaliana*.

## 3 General characteristics of SEC14L-PITPs

**Figure 2:**
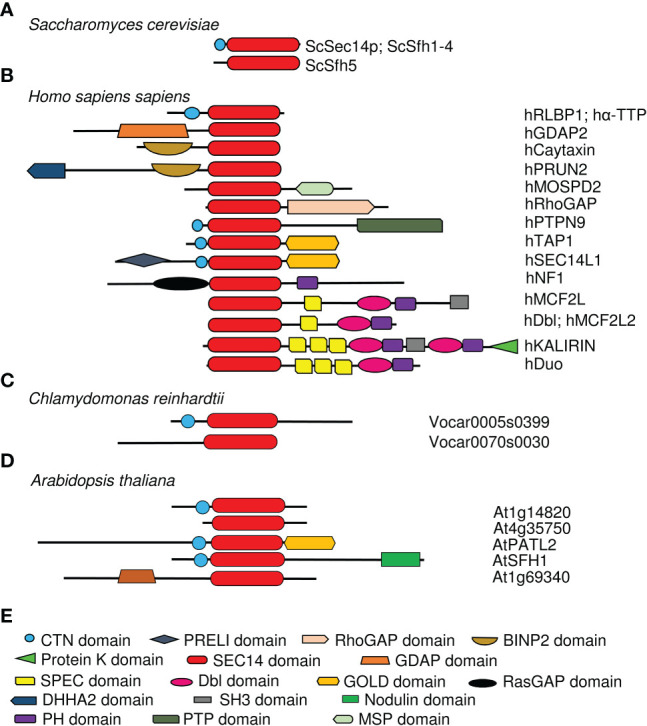
Schematic structures of representative SEC14L-PITPs showing the differing complexity between single- and multi-domain SEC14L-PITPs in unicellular and multicellular species. SEC14-PITPs in **(A)**, yeast *Saccharomyces cerevisiae*; **(B)**, *Homo sapiens*, **(C)**, *Chlamydomonas reinhardtii*; **(D)**, *Arabidopsis thaliana*. The presence of different types of domains is indicated in colors, and example names are provided on the right. **(E)** Symbols used in **(A–D). **Unicellular eukaryotes have simple single-domain SEC14L-PITPs, while multicellular eukaryotes have single- and various multi-domain proteins with various types of additional domains attached. Some multidomain proteins have independently evolved in the animal and plant lineage, e.g. SEC14-GOLD domain proteins, while others are unique in one of the lineages, e.g. SEC14-nodulin proteins in land plants.

The SEC14 domain forms a characteristic hydrophobic phospholipid-binding pocket at its carboxy (C)-terminus (Sha et al., 1998). Yeast Sec14p (304 AA) is the prototype for the SEC14 domain ([37-279AA] 12x α - helices, 6x β-strands, 8x 3_10_-helices; 2x distinct domains) (Sha et al., 1998) and was initially identified in a screen for secretory mutants (termed “SEC”) (Novick et al., 1980). An identical phospholipid-binding pocket was observed in several mammalian proteins, including the CELLULAR RETINAL-BINDING PROTEIN (CRALBP), TRIO and α-TOCOPHEROL-TRANSFER PROTEIN (α-TTP) (Crabb et al., 1998; Min et al., 2003). That is why the SEC14 domain is also known as CRAL-TRIO domain (Panagabko et al., 2003). A unique feature of the SEC14 domain is that the lipophilic ligand is bound and enclosed as a whole molecule in the hydrophobic lipid-binding pocket (Min et al., 2003; Schaaf et al., 2006), while other lipid-binding domains, like FYVE or PH, only bind lipid headgroups (Stahelin, 2009). The alpha helical amino (N)-terminus of Sep14p is defined as CRAL-TRIO-N-terminal extension (CTN) (IPR011074) and cannot be identified in all SEC14L-PITPs (Saito et al., 2007a). The ability to open and close the SEC14 lipid-binding pocket by structural changes seems to be essential for domain activity and the biological function of this domain (Ryan et al., 2007; Schaaf et al., 2008; Schaaf et al., 2011; Kono et al., 2013). The open status is believed to be the membrane-attached structure, while the closed conformation, the one binding a substrate, is understood as the cytosolic version of the SEC14 domain (Tripathi et al., 2014). This fits the observation that the CTN-SEC14 module is crucial for membrane association of SEC14L-PITPs, since loss of the module leads to the accumulation of the protein in the cytosol (Skinner et al., 1993; Sirokmany et al., 2006; Sun et al., 2006; Saito et al., 2007a; Saito et al., 2007b; Montag et al., 2020). The lipid-presentation model of SEC14L-PITP function is based on results indicating that the SEC14 domain is essential for promoting membrane trafficking by supporting PI(4)P-OH kinase activity (Strahl and Thorner, 2007) (**Figure 1B**). For example, the SEC14 domain can recognize membrane-bound phosphatidylcholine (PC) and present PI bound in the lipid-binding pocket of the SEC14 domain to a PI(4)P-OH kinases for phosphorylation, as proposed for Arabidopsis SEC14 protein named SFH1 (**Figure 1C**), according to (Kf de Campos and Schaaf, 2017). Phosphorylation of SEC14 protein may regulate the association of SEC14 proteins with membranes (Suzuki et al., 2016) (**Figure 1C**). It is remarkable that unicellular organisms have SEC14-only single-domain SEC14L-PITPs, whereas multicellular organisms of the animal and plant lineage have single-domain and multi-domain SEC14L-PITPs (**Figures 1D**, **2**) (Montag et al., 2020). The SEC14 domain represents a hydrophobic pocket-like lipid-binding site, in which a hydrophobic lipid substrate can bind (Sha et al., 1998). This site was found to be flanked by helical regions termed the anchor helix and gate helix. The anchor helix confers association with the membrane. The amphipathic gate helix keeps the lipid-binding site in an open or close conformation (Sha et al., 1998; Schaaf et al., 2008; Sugiura et al., 2021; Hornbergs et al., 2022; Yao et al., 2023) (**Figure 1E**). Additional residues may favor orientation either towards the negatively charged membrane or the cytosol, and they may also steer specificity for the lipid substrate (Sha et al., 1998; Schaaf et al., 2008; Sugiura et al., 2021; Hornbergs et al., 2022; Yao et al., 2023). Upon a heterotypic lipid exchange, an intermediate with two different lipophilic substrate-binding sites can form (Schaaf et al., 2008).

While the functions of several single-domain SEC14-only proteins are studied well, only a few multi-domain SEC14L-PITPs are characterized. At the same time, their abundance in higher eukaryotes highlights their importance. This raises several fundamental questions: Which are the pathways that single- and multi-domain SEC14L-PITPs are integrated in? What are the functions of the different domains within the multi-domain SEC14L-PITPs? Which protein-protein and protein-ligand interactions are relevant for the functions of SEC14L-PITPs? Herein, we review SEC14L-PITPs and focus on structure and function of single- and multi-domain SEC14-PITPs, especially in plants. We highlight particularly the subgroup of SEC14-GOLD proteins. We analyze which role might be played by subdomains to allow binding with phospholipids of the membrane and protein-protein interaction.

## 4 Single-domain SEC14-only PITPs

In addition to Sec14p and its homologs in yeast (Bankaitis et al., 1990; Cleves et al., 1991; Schnabl et al., 2003; Griac, 2007), single-domain SEC14-only proteins can be identified in Chlamydomonas and in higher eukaryotes, either with or without CTN (**Figure 2**) (Saito et al., 2007a; Montag et al., 2020). While all SEC14-only proteins in yeast are well characterized by demonstrating their roles in different aspects of the phospholipid metabolism, for example organization of the actin cytoskeleton, activation of phospholipase D or prevention of saturated fatty-acid accumulation (Li et al., 2000; Desfougeres et al., 2008; Yakir-Tamang and Gerst, 2009), only few SEC14-only proteins have been studied till now in higher multicellular eukaryotes. In spite of this, the studies of human SEC14-only proteins have increased the knowledge about the functions of SEC14L-PITPs and their essential roles within organisms. For example, human CRALBP is able to transport 11-cis retinaldehyde, the photosensitive component of rhodopsin, in its SEC14 lipid-binding pocket (Crabb et al., 1998; Fishman et al., 2004). This feature makes CRALBP essential for photoreceptor function. Mutations in its SEC14 domain can be the causes of neurodegenerative diseases affecting the eyesight by photoreceptor involution (Maw et al., 1997; Burstedt et al., 2001; Eichers et al., 2002). Another important human SEC14-only protein is α-TTP found to be most abundant in liver cells, where it is involved in vitamin E secretion, especially as α-tocopherol (α-Toc), but it is also expressed in mammalian uterine and placental cells during embryogenesis (Sato et al., 1993; Arita et al., 1997; Miller et al., 2012). Vitamin E is known to be an important antioxidant neutralizing reactive oxygen species (ROS) and radical formation involving membrane lipids (Nukala et al., 2018). Important for the biological function and localization of α-TTP is its ability to not only bind α-Toc (in its lipid-biding pocket) but also PIPs (at the entrance of the lipid-binding pocket) (Kono et al., 2013; Chung et al., 2016). PIP binding mediates the release of α-Toc at membranes by inducing the conformational change of the SEC14 binding pocket from closed to open (Meier et al., 2003; Kono et al., 2013). Mutations in α-TTP lead to the neurodegenerative disease AVED (ataxia, with vitamin E deficiency) caused by dramatic vitamin E deficiency, which results in disturbance of muscle activity (Ouahchi et al., 1995; Min et al., 2003). These two examples of human single-domain SEC14-only proteins show their critical roles in binding and transporting additional lipophilic ligands besides PI, PIPs and PC. Generally, the presence of SEC14-only proteins in unicellular and multicellular eukaryotes demonstrates the importance of regulating lipophilic-substance transport within a cell.

Out of the 15 described single-domain SEC14-only proteins of *A. thaliana*, a functional role and structural characteristics are known for CPSFL1, also known as PITP7 (**Figure 2**; **Table 1**) (Montag et al., 2020). This chloroplast-localized single-domain SEC14 protein is involved in formation of vesicles from the inner thylakoid membrane, where it may transfer phosphatidic acid and PIPs (Hertle et al., 2020; Kim et al., 2022). Interestingly, a similar single-domain SEC14 protein function was described for *Chlamydomonas reinhardtii*, where CPSFL1 defects also caused chloroplast dis-functioning, light sensitivity and low carotene contents in plastoglobuli and eyespot (García-Cerdán et al., 2020). Chlamydomonas CPSFL1 is able to bind besides phosphatidic acid also carotene and precursor substrates (García-Cerdán et al., 2020). Hence, this CPSFL1 single-domain SEC14 proteins seem to function in the development of chloroplasts by transporting and transferring relevant lipophilic substances inside chloroplasts.

**Table 1:** Selected *Arabidopsis thaliana* SEC14L-PITPs and their functions.

protein names	gene ID number	additional domains (other than SEC14)	protein-ligand interactions	protein-protein interactions	cellular roles	physiological effects	references
SFH1/ COW1	At4g34580	CTN, nodulin	PC, PI, PIP	oligomers	plays role in polarizing root hairs; rice orthologs OsSNDP2 and *OsSNDP3* play a role in polar tip growth of pollen	essential for root hair elongation; rice orthologs OsSNDP2 and *OsSNDP3* important during pollen cell elongation	Bohme et al., 2004; Vincent et al., 2005; Preuss et al., 2006 Huang et al., 2013; Ghosh et al., 2015; Moon et al., 2022
SFH5	At1g75370	CTN, nodulin	PA		transport of phosphatidic acid from ER to chloroplast	Chloroplast functioning	Yao et al., 2023
SFH7	At2g16380	CTN, nodulin	PA		transport of phosphatidic acid from ER to chloroplast	Chloroplast functioning	Yao et al., 2023
PATL1	At1g72150	CTN, GOLD	PI, PIPs	CaM4; SOS1; AMSH3; EXO70A1	plays a role in membrane trafficking; regulator of CaM4 and SOS1	plays a role in plant tolerance to abiotic stress and plant development	Peterman et al., 2004; Isono et al., 2010; Tejos et al., 2017; Chu et al., 2018; Zhou et al., 2018
PATL2	At1g22530	CTN, GOLD	PI, PIPs, α−Toc	IRT1; AMSH3; MPK4; EXO70A1	prevents membrane damage; plays a role in ROS prevention, iron acquisition and membrane trafficking	plant tolerance to abiotic stress, iron acquisition, plant development	Suzuki et al., 2016; Tejos et al., 2017; Wu et al., 2017; Montag et al., 2020; Hornbergs et al., 2022
PATL3	At1g72160	CTN, GOLD	PIPs	EXO70A1; AMV viral movement protein		plays a role in plant development; inhibits alfalfa mosaic virus infection	Peiro et al., 2014; Tejos et al., 2017; Wu et al., 2017
PATL4	At1g30690	CTN, GOLD		EXO70A1		plays a role in plant development	Tejos et al., 2017; Wu et al., 2017
PATL5	At4g09160	CTN, GOLD				plays a role in plant development	Tejos et al., 2017
PATL6	At3g51670	CTN, GOLD	Tomato homolog: α−Toc	EXO70A1; AMV viral movement protein		plays a role in plant development; inhibits alfalfa mosaic virus infection; a tomato homolog is required for chloroplast functioning	Peiro et al., 2014; Tejos et al., 2017; Wu et al., 2017; Bermudez et al., 2018
CPSFL1/PITP7	At5g63060	CTN	PA, PIPs		vesicle budding at inner thylakoid membrane in chloroplast	Chloroplast functioning	Hertle et al., 2020; Kim et al., 2022

α-Toc, α-tocopherol; CTN, CRAL-TRIO N-terminal extension; GOLD, Golgi dynamics; PA, phosphatidic acid; PC, phosphatidylcholine; PI, phosphatidylinositol; PIP, phosphoinositide/ phosphoinositol phosphate; SEC14L-PITP, SEC14-like phosphatidylinositol transfer protein.

## 5 Multi-domain SEC14L-PITPs

The number and modular complexity of SEC14L-PITPs increases in multicellular eukaryotes (**Figure 2**) (Montag et al., 2020). Through the presence of one or more additional domains the functions of SEC14L-PITPs are extended, the functions of different domains are better coordinated with respect to each other in a cell and upon loss of function the risk of second-site dominant effects is reduced (**Figure 1D**). Multi-domain SEC14L-PITPs are not only regulators of lipophilic substance transport but they may have the ability to function, e.g. as proteins with enzymatic functions, guanine exchange factors (GEFs) or GTPase-activating proteins (GAPs). For example, the human multi-domain SEC14L-PITP TYROSINE-PROTEIN PHOSPHATASE NON-RECEPTOR TYPE 9 (PTPN9) has an additional protein phosphatase catalytic (PTP) domain (IPR000242) and functions as a tyrosine phosphatase (Denu and Dixon, 1998). The CTN-SEC14 module of PTPN9 is responsible for protein localization to the outer surface of secretory vesicles binding either phosphatidylserine (PS) or PI(3,4,5)P_3_ (and other PIPs) (Kruger et al., 2002; Krugmann et al., 2002; Huynh et al., 2003; Zhao et al., 2003; Saito et al., 2007a; Saito et al., 2007b). The membrane targeting function of the (CTN)-SEC14 domain and its role in intracellular vesicle trafficking could also be recognized in human multi-domain SEC14L-PITPs functioning as GEFs and GAPs and thereby regulating the Ras/Raf-signaling pathway (Ueda et al., 2004; Sirokmany et al., 2006; Sun et al., 2006). For example, human SEC14L-PITPs with a GAP function are KALIRIN, Dou or RhoGAP, while MCF2 or MCF2L are functioning as GEFs. Mutations in all these proteins are linked to neurodegenerative diseases or cancer. Defects in human NEUROFIBROMIN 1 (NF1), a putative negative regulator of the Ras-signaling pathway, are disease-associated, especially when occurring in the double domain structure of SEC14-PH (Rad and Tee, 2016). The Pleckstrin homology (PH) domain (IPR001849) has a phospholipid-binding specificity for PI(4,5)P_2_ and seems to be involved in protein recruitment to membranes (Hyvonen et al., 1995; Lemmon and Ferguson, 1998; Lemmon, 2007). Patients with an *NF1* mutation are developing the Recklinghausen disease/Watson syndrome and have a significantly increased cancer risk (Rasmussen and Friedman, 2000; D'Angelo et al., 2006; Yap et al., 2014; Peltonen et al., 2017). Another example of SEC14L-PITPs playing an important role in human health is the prostate cancer suppressor PROTEIN Prune Homolog 2 With BCH Domain (PRUNE2) containing the additional BINP2 domain and DHHA2 domain (IPR004097) at its N-terminus (Salameh et al., 2015). The human multi-domain SEC14L-PITP GANGLIOSIDE-INDUCED DIFFERENTIATION-ASSOCIATED PROTEIN 2 (GDAP2) contains a GDAP macro domain (IPR035793) at its N- terminus, which possibly binds ADP-ribose, and is localized to the lysosomal membrane (Martzen et al., 1999; Hassa et al., 2006). Its exact function is unknown, but recently homologs were identified in plants, suggesting a conserved function (Montag et al., 2020).

### 5.1 SEC14-nodulin proteins (plant-specific)

A plant-specific subfamily of multi-domain SEC14L-PITPs are SEC14-nodulin proteins exhibiting an additional C-terminal nodulin domain, present in this combination in seed plants (**Figure 2**; **Table 1**) (Kapranov et al., 2001; Denancé et al., 2014; Montag et al., 2020). The nodulin name is derived from nodulin genes and proteins highly expressed and accumulating during the nitrogen-fixing symbiosis of legume plants at the root hair invasion, infection and nodule developmental and nitrogen-fixing site (Denancé et al., 2014). It was therefore very interesting to find that one function of SEC14-nodulin proteins is to be basic regulators in polarizing membrane trafficking (Vincent et al., 2005; Huang et al., 2013; Ghosh et al., 2015). A well-studied member of this protein family in this context is *A. Thaliana* SFH1, also known as CAN OF WORMS1 (COW1), which is involved in root hair biogenesis by controlling the tip-directed gradient of PI(4,5)P_2_ (and PI(4)P) (Bohme et al., 2004; Vincent et al., 2005; Preuss et al., 2006; Ghosh et al., 2015). *Sfh1* loss-of-function mutant plants have short root hairs defective in elongation (Ghosh et al., 2015). Essential for this are both the SEC14 domain and the nodulin domain including the C-terminal poly-lysine motif stretch of the nodulin domain (Ghosh et al., 2015). The SEC14 domain lipid-binding activity is important, since mutations leading to reduced phosphatidylinositol transfer *in vitro* do not complement the root hair phenotype of *sfh1* mutants (Huang et al., 2016). The poly-lysine stretch is presumably required for PI(4,5)P_2_ binding and assembly of the SEC14-nodulin protein at the plasma membrane since mutants devoid of such a stretch do not complement the *sfh1* phenotype, do not locate at the plasma membrane in yeast cells and have an altered oligomerization behavior. According to the working model proposed by Ghosh et al., (2015), oligomers of SFH1 present PI contained inside the SEC14 domains at membrane sites to phosphatidylinositol-4 or -5-phosphate kinase, thereby changing the phosphatidylinositol landscape and properties of the plasma membrane. Subsequently, oligomeric SFH1 complexes may form at the plasma membrane. These events may trigger root hair cell elongation. A similar function is proposed for rice OsSNDP2 and *OsSNDP3* during pollen tube elongation (Moon et al., 2022). Two further SEC14-nodulin proteins from Arabidopsis have been recently studied in physiological contexts, which are SFH5 and SFH7 (Yao et al., 2023). These two proteins were found to localize at ER and chloroplast membranes. SFH5 and SFH7 were able to bind phosphatidic acid and transfer it between membranes *in vitro*. Additionally, it was shown that double knockout mutants had aberrant thylakoid membrane structures in chloroplasts. Interestingly, phosphatidic acid is the precursor to several lipids produced in chloroplasts and required for thylakoid assembly, and the *sfh5 sfh7* double mutants had reduced amounts of such lipids. Hence, these findings strongly suggest that the SEC14-nodulin proteins SFH5 and SFH7 mediate the transport of phosphatidic acid from ER to chloroplast perhaps at interorganellar contact sites (Yao et al., 2023). SEC14-nodulin proteins therefore play roles in different organs (roots and leaves) and are involved in different processes requiring lipid transfer, such as polarity control and organellar contacts.

### 5.2 SEC14-GOLD proteins

**Figure 3:**
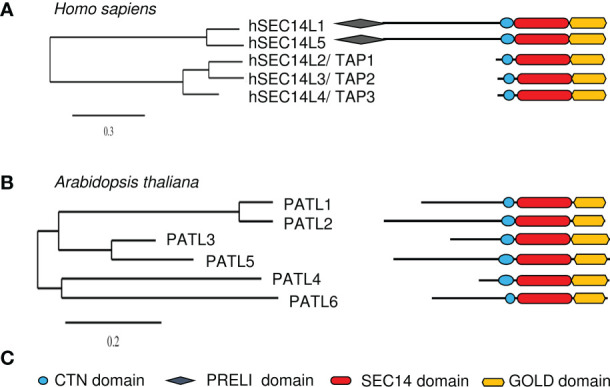
Overview of human and *Arabidopsis thaliana* SEC14-GOLD proteins. Phylogenetic trees of **(A)**, human and **(B)**, Arabidopsis SEC14-GOLD proteins and their modular architecture. [Phylogenetic analysis, domain identification and alignments were performed as described in Montag et al., 2020]. **(C)**, Symbols used in **(A, B)** Plant PATLs have differing N-terminal regions, while human SEC14-GOLD proteins have either no extensive N-terminal region or a N-terminal region with PRELI domain. CTN, cellular retinal-binding protein and TRIO protein N-terminal extension; PRELI, PRELI/MSF resembling human PRELI protein; SEC14, secretory mutant 14 protein; GOLD, Golgi dynamics.

A well-studied family of multi-domain SEC14L-PITPs is the SEC14-GOLD family. All SEC14-GOLD proteins contain a Golgi dynamics (GOLD) domain (IPR009038) at their C-terminus (**Figures 2**, **3**; **Table 1**). They can be found in insects and vertebrates. In the green lineage, SEC14-GOLD proteins were identified in bryophytes *Marchantia polymorpha* and vascular plants (Montag et al., 2020). It has been shown that the GOLD domain functions in membrane trafficking along the secretory pathway by mediating diverse protein-protein and protein-membrane interactions (Sohda et al., 2001; Anantharaman and Aravind, 2002; Carney and Bowen, 2004; Pastor-Cantizano et al., 2016; Pastor-Cantizano et al., 2018). The GOLD domain is either present in single-domain proteins or co-occurs with other domains, which are all involved in lipid-binding (Anantharaman and Aravind, 2002; McPhail et al., 2017; Pastor-Cantizano et al., 2016).

The analyses of SEC14-GOLD proteins revealed two subgroups of this family in humans (**Figure 3A**). Next to the two defining domains, SEC14-LIKE1 (SEC14L1) and SEC14-LIKE5 (SEC14L5) have an additional N-terminal PRELI/MSF1 domain (IPR009038/IPR006797) (Anantharaman and Aravind, 2002). Similar SEC14-GOLD proteins with additional PRELI domain can also be identified in other higher eukaryotes like Zebrafish*, Drosophila melanogaster*, *Mus musculus*, and *Caenorhabditis elegans*, but not in any of the checked plant and yeast species. It is assumed that the PRELI/MSF1 domain could function in protein association to membranes as well as in transferring lipids (Anantharaman and Aravind, 2002; Yu et al., 2015), due to its involvement in mitochondrial protein sorting and phosphatidylethanolamine metabolism (Nakai et al., 1993; Hall et al., 2011). For example, hSEC14L1 is able to associate with two transporters, the VESICULAR ACETYLCHOLIN TRANSPORTER (VACht) and the CHOLINE TRANSPORTER 1 (CHT1), on synaptic vesicles (Ribeiro et al., 2007). This indicates that hSEC14L1 may play a fundamental role in intracellular vesicle trafficking (Ribeiro et al., 2007). Additionally, it has a negative regulatory function on RETINOIC ACID-INDUCIBLE GENE I (RIG-I), important for antiviral immunity response. RIG-I interaction with other proteins is inhibited through its interactions with hSEC14L1, via its PRELI domain and SEC14 domain (Li et al., 2013). hSEC14L5 was characterized as a potential target for post-traumatic stress disorder (PTSD) found in a study to identify molecular and genetic key players in this disease (Chitrala et al., 2016). Human TOCOPHEROL-ASSOCIATED PROTEINs (TAPs), TAP1 (SPF/SEC14L2), TAP2 (p45/SEC14L3) and TAP3 (SFP2/SEC14L4), have a SEC14-GOLD domain combination with no additional N-terminal extensions (termed “N region”) (**Figure 3A**). They have the ability to bind α-Toc, α-Toc derivatives, squalene, phosphatidylglycerol, phosphatidylcholine (PC), PI and PIPs (Chin and Bloch, 1985; Kempna et al., 2003; Stocker and Baumann, 2003). TAP proteins have a Rab-like small GTPase activity (Habermehl et al., 2005; Gong et al., 2017). Furthermore, the ability to bind α-Toc and its derivatives indicates a role of human TAPs in preventing lipid peroxidation. This is supported by the observation that phosphorylated hTAP1 is able to stimulate cellular cholesterol biosynthesis, since protecting low density lipoproteins from oxidation may inhibit cholesterol uptake (Neuzil et al., 1997; Shibata et al., 2001; Stocker and Baumann, 2003). Another hint to that assumption is that the presence of hTAP1 is able to increase vitamin E-mediated membrane protection from lipid peroxidation, which positively influences RNA replication of the hepatitis C virus in cell cultures (Saeed et al., 2015; Li et al., 2018). Additionally, hTAP1 and its functional orthologue *Cgr-1* in *C. elegans* are playing a conserved role in the Ras/Raf pathway by being regulators of the Raf-signal activation and thereby suppressing its oncogenic capacity (Johnson and Kornfeld, 2010). Here again its ability to bind α-Toc positively influences health by regulating the uptake of α-Toc into cancer cells to stop cell growth and amplification. But hTAP1 is not only involved in tumor suppression by mediating α-Toc uptake and lipid protection, it also contributes to the regulation of PI(3)P Kinase γ (PI3Kγ) activity, either by blocking its subunit interaction or starting its activity, which then leads to VASCULAR ENDOSOMAL FACTOR (VEGF) expression (Ni et al., 2005; Wang et al., 2009; Zingg et al., 2014; Zingg et al., 2015; Zingg et al., 2017). Another fact linking hTAP1 to carcinogenesis is the observation that it is highly expressed in breast and prostate tissue, but downregulated in prostate and breast cancer cell lines, as well as in human breasts with invasive breast carcinomas (Ni et al., 2005; Wang et al., 2009). In zebrafish, TAP2 is crucial for the hydrolysis of PI(4,5)P_2_ by phospholipase C (Gong et al., 2017). Rat (*Rattus norvegicus*) p45, a hTAP2 homolog, especially binds PI(3,4,5)P_3_ *in vitro* and localizes with it in secretory vesicles, the cytoplasm and the extracellular space (Merkulova et al., 2005). Deletion of the SEC14 domain leads to inhibited secretion into the extracellular space, indicating that the SEC14 domain is essential for secretion (Merkulova et al., 2005). The expression of rat SPF2, a homolog of hTAP3, is mainly observed in skin and respiratory tissue (Merkulova et al., 1999; Kempna et al., 2003). Recombinant SPF2 is able to stimulate the monooxygenase but not as efficient as TAP1 (Mokashi et al., 2004). Its activity is thereby stronger dependent on regulation by protein kinase A phosphorylation, guanine nucleotides and α-Toc, than TAP1 (Mokashi et al., 2004). An alternative splicing pattern was obtained for human TAP3 (Kempna et al., 2003). Due to this, reduced levels of biologically active hTAP3 could increase the risk of disease outbreak associated with the secretory capability of tissues/cells (Zingg et al., 2008; Kempna et al., 2010), underlining possible roles of TAPs as tumor suppressors. Thus, the data on animal SEC14-GOLD proteins demonstrate SEC14-GOLD protein roles in intracellular vesicle trafficking by interaction with PIPs. Additionally, it shows their function as negative regulators via protein-protein interactions and demonstrates their oncogenic role. Furthermore, the data highlights their possible function as tumor suppressers, e.g. by mediating vitamin E transport and by preventing cellular damage by ROS and radicals.

**Figure 4:**
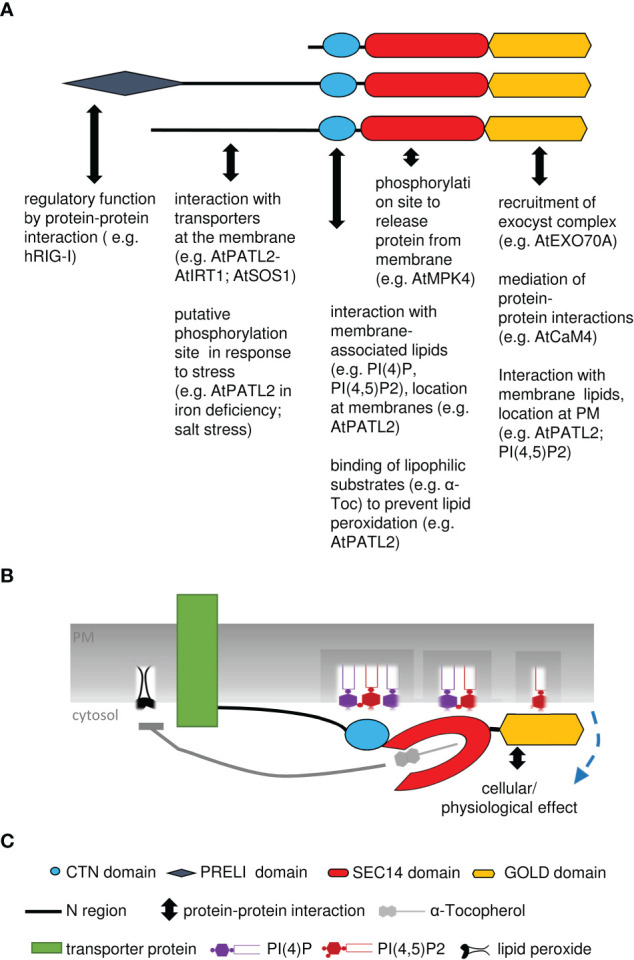
Summary model of SEC14-GOLD protein functions. **(A)**, Summary of various interactions with proteins, lipids or ligands, described for different parts of SEC14-GOLD proteins. **(B)**, Model of AtPATL2, binding to phospholipids and interacting with transport protein in the plasma membrane, leading to prevention of oxidative stress and lipid peroxidation; PATL2 may exchange or present the antioxidant a-tocopherol. The cellular physiological effect may comprise vesicle formation and regulation of transporter abundance and activity. **(C)**, Explanations of symbols used in **(A, B)**.

In Arabidopsis and other plants, SEC14-GOLD proteins are called PATELLINs (PATLs), named after patella, the Latin word for small plate, referring to PATL1 localization at the developing cell plate (Peterman et al., 2004). Analysis of the SEC14L-PITP superfamily in Arabidopsis revealed six PATL proteins, with a CTN-SEC14 and GOLD domain but no other N-terminal domains (**Figure 3B**; **Table 1**) (Peterman et al., 2004). PATLs display a variable N region of unknown structure, however different small motifs (coiled coil- and PXXP motifs) can be found (Peterman et al., 2004; Neduva and Russell, 2006; Diella et al., 2008; Montag et al., 2020). The N regions of PATLs vary in amino-acid sequences and are unique for each protein (Montag et al., 2020). PATLs, except of PATL6, show an overall acidic N region due to repeats of glutamate (E) (Peterman et al., 2004). But they also show a pattern of lysines (K) surrounding the E repeats in PATL1, PATL2 and PATL4 (Montag et al., 2020). Independently of the N regions, plant SEC14-GOLD proteins form three clades with subgroup-specific amino acid substitutions in the GOLD domain, which may define different functional categories (Peterman et al., 2006; Bermudez et al., 2018; Montag et al., 2020). Expression analyses of PATLs uncovered overlapping and clade-specific clusters, and together with studies on multiple knock-out plants, this indicates partial redundancy within the family (Tejos et al., 2017; Montag et al., 2020). Multiple *patl* mutants demonstrated the essential role of PATLs in Arabidopsis patterning and polarity by revealing auxin response phenotypes and developmental defects due to decreased polarization of the auxin transporter PIN-FORMED 1 (PIN1) (Tejos et al., 2017). The role of PATLs during plant development can be confirmed by the observation that *PATL1* expression was increased in developing leaves and vascular tissues and by cellular localization of PATL1 at the plasma membrane and the cell plate during cell division (Peterman et al., 2004). Distinct and overlapping localization patterns, were found for all other PATLs. PATLs are peripheral membrane proteins found to localize at the plasma membrane, at the cell plate and/or were found to be cytosolic. *PATL* genes were expressed in leaf epidermis cells, vascular tissues, during embryogenesis, during development of lateral-root primordia and during differentiation of the root apical meristem (Suzuki et al., 2016; Tejos et al., 2017; Wu et al., 2017). Protein localization during development and differentiation links PATLs closely to membrane trafficking supported through the observation that PATL1, PATL2 and PATL3 bind PIPs (Peterman et al., 2004; Suzuki et al., 2016; Wu et al., 2017). PATLs are involved in a number of different protein interactions at membrane sites (**Figure 4**). Although PATL1 preferentially binds PI(5)P, PI(3)P and PI(4,5)P_2_ and AtPATL3 mainly binds PI(4)P and PI(4,5)P_2_, both still have the ability to associate with all other PIPs (Peterman et al., 2004; Wu et al., 2017). All domains of PATL2 contributed to PIP association (Montag et al., 2020) (**Figure 4**). The CTN-SEC14 module of PATL2 was found to govern membrane association of the protein, and the GOLD domain to specify plasma membrane localization, presumably by recognizing PI(4,5)P_2_ maybe through its lysine motif (Montag et al., 2020). A hint linking PATLs to plasma membrane protein regulation and membrane trafficking is the observation that PATL1 and PATL2 were able to interact with plasma membrane proteins (Chu et al., 2018; Zhou et al., 2018; Hornbergs et al., 2022) (**Figure 4**). Through its GOLD domain PATL1 interacted with CALMODULIN-4 (CaM4), a multifunctional sensor for Ca^2+^, and via its N region it interacted with SALT OVERLY-SENSITIVE 1 (SOS1), a Na+/H+ antiporter localized at the plasma membrane (Chu et al., 2018; Zhou et al., 2018). Its closest homologue PATL2 interacted through its N region with IRON-REGULATED TRANSPORTER 1 (IRT1), an essential protein for iron acquisition by roots in soil (Hornbergs et al., 2022) (**Figure 4B**). PATL1 and PATL2 contribute to stress tolerance by affecting plant responses to cold, salt and iron nutrition-related stress. In addition, both seem to be involved in preventing damage caused by reactive oxygen species (ROS) and radicals (Zhou et al., 2018; Hornbergs et al., 2022). *patl2* loss-of-function mutants exhibited enhanced iron reduction activity in roots, a response required for iron acquisition via IRT1 in Arabidopsis, as well as enhanced lipid peroxidation phenotypes (Hornbergs et al., 2022). Interestingly, interactome analysis of tagged PATL2 retrieved ROS response/metabolism proteins and, under iron deficiency, endomembrane trafficking regulators. PATL2 protein was found to bind the antioxidant α-Toc in the lipid-binding SEC14 domain (Hornbergs et al., 2022). Vitamin E deficiency also caused iron utilization phenotypes. Taken together, PATL2 may recruit a ROS response interactome to IRT1 sites and present or transfer α-Toc to IRT1 membrane sites. Since IRT1 mediates uptake of reactive iron and other metal ions, vitamin E compounds may protect from potential oxidative stress due to lipid peroxidation catalyzed in the presence of these reactive Fenton metal ions. Subsequently, endomembrane trafficking may affect the regulation of IRT1 (Hornbergs et al., 2022). In this context, it is interesting to note that phosphorylation of the N region was identified under Fe deficiency (Lan et al., 2011) and during salt stress, in response to oligogalacturonides, and brassinosteroid signaling (Tang et al., 2008; Hsu et al., 2009; Chang et al., 2012; Mattei et al., 2016), indicating that protein interaction of PATL2 with IRT1 may be under control of protein phosphorylation of the interacting N region. PATL1 and PATL2 co-immunoprecipitated with ASSOCIATED MOLECULE WITH THE SH3 DOMAIN OF STAM3 (AMSH3), a deubiquitinating enzyme required for intracellular trafficking and vacuole biogenesis in Arabidopsis, next to other proteins with reported or expected function in intracellular trafficking processes (Isono et al., 2010). PATL2 is phosphorylated by MAP KINASE4 (MPK4) within the SEC14 domain, which might be important for the release of PATL2 from the membrane (Suzuki et al., 2016) (**Figure 4A**). Additionally, phosphorylation of the SEC14 domain of PATL2 could be detected after short- term cytokinin treatment and sugar stress (Niittyla et al., 2007; Cerny et al., 2011). This indicates that PATL2 might undergo dynamic post-translational regulation in response to plant stress. PATL3 recruitment to the plasma membrane depended on interaction of its GOLD domain with EXO70A1, a subunit of the exocyst complex participating in intracellular vesicle transport (He and Guo, 2009; Fendrych et al., 2013; Wu et al., 2017). Interestingly, all other PATLs except of PATL5, were able to interact with EXO70A1 (Wu et al., 2017). Moreover, PATL3 and PATL6 inhibited stem infection spread of the alfalfa mosaic virus by interfering with virus movement through interaction with a PLASMODESMATA TARGETING MOVEMENT PROTEIN (AMV MP) and thereby preventing subcellular targeting (Peiro et al., 2014) (**Figure 4A**). Tomato TOCOPHEROL BINDING PROTEIN (SlTBP) is a homologue of Arabidopsis PATL6 and clusters together with other plant PATLs due to a plastid-targeting signal (Bermudez et al., 2018). The *SlTBP* gene is mainly expressed in photosynthetic active tissues and the protein is localized to plastids. Its potential ability to bind α-Toc makes it a key player in controlling possibly vitamin E movement between plastids and the ER, which affects lipid metabolism within these organelle (Bermudez et al., 2018). Additionally, SlTBP is involved in maintaining chloroplast membrane structure, affecting its lipid profile (Bermudez et al., 2018). Interestingly, PATL1, PATL2 and PATL5 were identified as putative cargo receptors in proteomic studies searching for components of the chloroplast vesicle transport pathway. The proteins were identified localizing to the chloroplast envelope (Kleffmann et al., 2004; Ferro et al., 2010; Khan et al., 2013).

Taken together, PI/PIP and α-Toc binding, cell plate and membrane localization, phosphorylation, increased expression during stress responses, and interaction with membrane and trafficking proteins indicate that PATLs are basic cell regulators adapting the cell/organism during cell division and growth and to altered environmental influences and in response to external stimuli. As regulatory proteins PATLs may be involved in membrane trafficking, e.g., by initiating vesicle formation. Furthermore, they might play a role in protecting the cell from ROS and radical damage, by offering α-Toc as an antioxidant or through regulating the activity of membrane proteins.

## 6 Conclusion

The SEC14 domain is the common feature of SEC14 proteins. The domain is capable to recognize, bind, transport, and exchange single lipophilic molecules between membranes inside a lipid-binding site. Unlike other lipid-binding domains, the SEC14 domain not only binds phospholipids, but also other lipophilic substances, e.g., α-Toc, carotenoids. This ability leads to further cellular and physiological effects when these lipid transfer activities are required. This characteristic and the presence of SEC14L-PITPs in higher eukaryotes indicates a conserved function and highlights the need of controlling lipid signaling and membrane trafficking. Especially, the presence of additional domains in multi-domain SEC14L-PITPs of higher multi-cellular eukaryotes indicates an increasing variety of functions due to enhanced possibilities for protein interaction, cellular localization or enzyme activities. These additional domains could be involved in the sensing of the lipid environment at the membrane or in changes of lipid-signaling pathways leading to environmental adaptation. The idea is supported by the findings that multi-domain SEC14L-PITPs are able to influence cell division, vesicle formation, lipid signaling, environment responsiveness and organism development, which directly affects the organism fitness/health. The SEC14 domain is also of special interest, since mutations in the SEC14 domain result in defects in development and in the plant-stress response and tolerance, as well as in neurodegenerative diseases and an increased cancer risk in humans.

Not all complex tasks of SEC14L-PITPs are understood right now and many interesting questions remain to be answered. For example, are SEC14L-PITPs regulated by post- transcriptional regulation? What are the effects of protein phosphorylation? What is the function of the plant-specific regions of SEC14L-PITPs? How do SEC14L-PITPs influence protein activity and metabolic reactions? In which pathways do yet uncharacterized proteins of this kind play a role? Identifying the functions of SEC14L-PITPs on cellular levels can help to understand the adaptation of regulatory pathways to environmental changes.

## Author contributions

KM and PB wrote the article and prepared figures. All authors contributed to discussion. PB and RI revised the manuscript. All authors contributed to the article and approved the submitted version.
